# Low Cost Eye Tracking: The Current Panorama

**DOI:** 10.1155/2016/8680541

**Published:** 2016-03-13

**Authors:** Onur Ferhat, Fernando Vilariño

**Affiliations:** ^1^Computer Vision Center, Edifici O, Campus UAB, 08193 Bellaterra, Spain; ^2^Computer Science Department, Universitat Autònoma de Barcelona, Edifici Q, Campus UAB, 08193 Bellaterra, Spain

## Abstract

Despite the availability of accurate, commercial gaze tracker devices working with infrared (IR) technology, visible light gaze tracking constitutes an interesting alternative by allowing scalability and removing hardware requirements. Over the last years, this field has seen examples of research showing performance comparable to the IR alternatives. In this work, we survey the previous work on remote, visible light gaze trackers and analyze the explored techniques from various perspectives such as calibration strategies, head pose invariance, and gaze estimation techniques. We also provide information on related aspects of research such as public datasets to test against, open source projects to build upon, and gaze tracking services to directly use in applications. With all this information, we aim to provide the contemporary and future researchers with a map detailing previously explored ideas and the required tools.

## 1. Introduction

From a computer scientist's perspective, human beings are machines which receive input from their sensors such as ears, eyes, and skin and which interact with the world they live in through their actuators, which are their hands, feet, and so on. Their attention can be understood by analyzing the way they direct their sensors (i.e., looking at specific locations or inspecting unknown objects by touching or smelling). Moreover, as in the case of robots, examining this attention can give us hints about their state of mind and their way of reasoning.

Among the human senses, sight has an important place in today's world where we are surrounded with digital displays be it in our mobile phones, our computers, or televisions. Instead of making passive observations of the objects around, it also gives hints about what the person actively chooses to see through eye movements. Analysis of these movements, therefore, sparked great interest in research communities.

Devices or systems that track a person's eye movements are called eye trackers or gaze trackers. Currently, the most widespread techniques used in these trackers make use of light sources and cameras that operate in the infrared (IR) spectrum. There are many available commercial models that are in the form of either eyeglasses or table mounted devices [1–3] and also open source alternatives that allow the use of custom hardware [4].

Visible light gaze tracking, on the other hand, does not require any special hardware and aims to solve the task making use of regular cameras. In this paper, we will concentrate on this class of trackers and survey the related research. Furthermore, we will limit our search to the table mounted setup (also called remote setup) as it is ubiquitous in contemporary devices and it removes the restrictions for camera placement (with a few exceptions). Our aim and contribution is as follows:

- To provide an exhaustive literature review.
- To comment on these works from various perspectives.
- To list publicly available datasets.
- To list open source software.
- To list gaze trackers as a web service.

The rest of the paper is organized as follows: we will start with an overview of the software structure used in remote, visible light gaze trackers. Then, we will categorize and explain the previous work according to the techniques used and continue with two other categorization schemes: how/if they are calibrated and how/if they handle head movements. Afterwards, we will list and comment on the available datasets, online gaze tracking services, and open source projects. We will finish with our conclusions regarding the current state and future directions.

## 2. Categorization and Structure of Visible Light Gaze Trackers

The categorization of the works that we analyze in this paper is not trivial, because the borders between groups of methods are not always clear and in the literature different naming schemes exist.

In the early review by Morimoto and Mimica [5], methods using the eye appearance (i.e., eye region image pixels) directly for gaze estimation are called appearance-based or view-based methods, and the rest is left unnamed. Here, the given name refers to all the visible light methods and does not give information about the subcategories. Even in a more recent survey [6] where both infrared (IR) and visible light methods are considered, the latter group is considered as just an alternative, and its subcategories are left unclear. Other categorization schemes also build on this ambiguity: appearance-based versus feature-based [7, 8] and appearance-based versus model-based [9, 10]. It should also be noted that the “appearance-based” name is still being used to refer to all visible light methods [11, 12], adding to the confusion.

With the aim of clearly identifying the borders between different visible light gaze estimation techniques (and hopefully not adding to the confusion), we present a new categorization scheme:

1. *Appearance-Based*. These methods only use the eye image pixel intensities to create a mapping to the gaze estimation. The image pixels are converted to a vector representation via raster scanning and fed to the estimation component.
2. *Feature-Based*. Methods of this category also make use of a mapping to calculate the gaze; however, they use richer feature vectors compared to the methods in the previous category (i.e., not just pixel intensities).
3. *Model-Based*. Compared to the discriminative approach of the first two categories, the methods belonging to this category follow a generative approach by trying to model the eyes and maybe even the face. The gaze is calculated geometrically using the model parameters.

**Figure 1:**
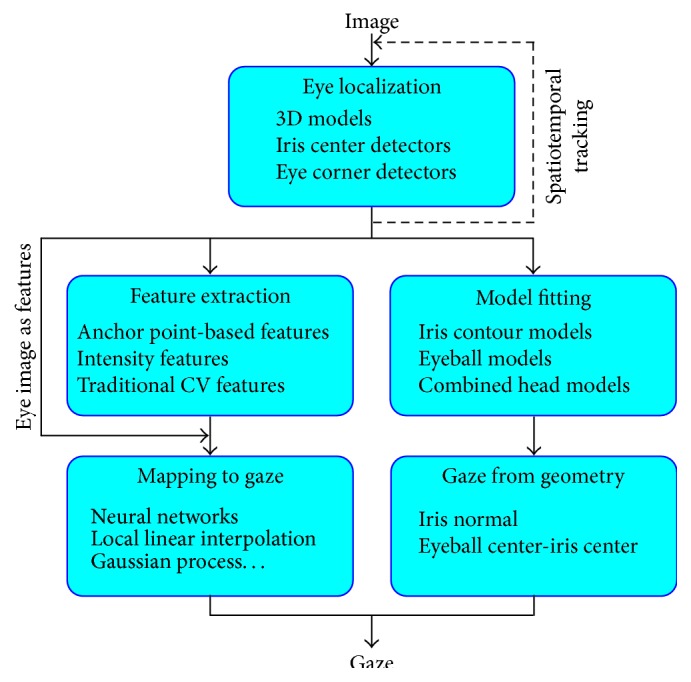
The common software structure for visible light gaze trackers. The methods start by locating the eyes. To make the estimation more stable, spatiotemporal tracking may be utilized at this step. Later, the location information is used to extract features, fit 2D or 3D models to the eyes, or just extract the eye region image. In the case of model-based methods, the fitted model is used to calculate the gaze geometrically, whereas in the other methods, a mapping function is necessary to calculate the gaze angle or point.

After explaining our categorization and the reasoning behind it, we can continue with the discussion about the software pipeline of these trackers. Although the variation in details is huge, a common skeletal structure that describes their software implementation can easily be identified as seen in Figure 1.

The input to the system is generally a video stream; however, examples of systems working on still images are also found [13]. In the former case, the previously processed video frames' results may be used to improve the performance for the next frames [14].

The first task in the pipeline is to extract the eye region. If an optional head pose estimation component is present, and if its output contains information about the eye location, it may be used directly as the location or it may be used as a rough initial estimate for the actual eye locator. Otherwise, the eye locator component has the option of using face detectors to restrict the processed image area and reduce computational cost [15, 16]. In order to calculate accurate eye location, the system can make use of iris center detectors [17], eye corner detectors [18], or 3D eye models that take into account the appearance of the entire eye [19].

Once the region of interest (ROI), that is, the eye region, is located, the second step is to prepare the input for the gaze estimation component. Depending on the class of gaze estimation method, the required input for the last step varies. In* appearance-based* methods, the extracted eye image from the first step is used directly as the input. Here, each image pixel intensity is considered as one dimension of the input vector. As the change in illumination and shadows may interfere with these inputs, this class of methods may not always give robust results.

*Feature-based* methods try to break the direct connection between the raw pixel intensities and the final input vector, in an attempt to increase robustness to lighting changes. Some of the features used in the literature are as follows:

- Pixel positions of keypoints (e.g., inner eye corners, iris center, and eyelid) [20, 21].
- Their relative positions (i.e., vectors connecting two positions) [22–24].
- Standard computer vision features such as histogram of oriented gradients (HOG) [25, 26] and local binary patterns (LBPs) [11, 27].
- Features calculated by a convolutional neural network (CNN) [13].
- Features grouping and summarizing pixel intensities [28–31].

Finally, the* model-based* gaze estimation methods require the parameters for a 2D or 3D eye model as the input. In case of 2D, these can be the parameters defining the iris edge model [32]; in the 3D case, it can get more complex to include 3D positions of the eyeball center [33] or other facial landmarks [34].

The last step in the described pipeline is the estimation of the gaze, given the inputs calculated in the previous step. Appearance-based and feature-based methods require a mapping function that maps the input vectors to the gaze point or the gaze direction. The commonly used techniques include neural networks (NNs) [35, 36], Gaussian process (GP) regression [14, 37], and linear interpolation [38, 39]. On the other hand, model-based methods use the geometry of their 3D model (e.g., normal vector for the iris of 3D eye ball model) to calculate the gaze [40, 41].

## 3. Methods for Single Camera Remote Gaze Tracking

In this section, we categorize the works that we focused on according to our scheme. A summary of these works can be seen in Table 1.

### 3.1. Appearance-Based Methods

The first techniques proposed for visible light gaze tracking introduced the category of appearance-based methods [16, 35, 42]. These methods are characterized by their use of eye image pixel intensities as their features for gaze estimations. After a possible histogram normalization step for standardizing image appearances over the whole dataset, these feature vectors are fed to the estimation component which maps them to screen coordinates.

#### 3.1.1. Neural Networks

One of the most popular mapping functions used in eye tracking is neural networks (NNs). In their pioneering work, Baluja and Pomerleau [35] introduce the first method making use of NNs. They test their system extensively by varying the inputs (iris region or entire eye), NN structure (single continuous or divided hidden layer), and the hidden layer unit number. In another experiment, they demonstrate that, by training the system with inputs from different head poses, the system can even handle small head movements. Finally, they top their system with an offset table that is used to correct the systematic shifts in actual eye tracker use. In the best case, their reported accuracy is around 1.5°.

Stiefelhagen et al. [16] use skin color segmentation and pupil detection to replace the use of a light source for this task in the original work of Baluja and Pomerleau. Xu et al. [42] introduced an iterative thresholding method to locate the iris region accurately and also proposed Gaussian smoothing for outputs of the NN during training. Two recent works [43, 44] used the NN technique for gaze tracking on commercial tablet computers and report lower accuracy (average error > 3°), mainly because of the low sampling rates in tablets and high training data demand of the NNs.

#### 3.1.2. Local Linear Interpolation

A recently more popular alternative to NN mapping is local linear interpolation as proposed for gaze tracking by Tan et al. [38]. In their work, they see the eye region images as coming from an appearance manifold, and gaze estimation is posed as a linear interpolation problem using the most similar samples from this manifold. Although this work makes use of IR illumination for eye localization, the gaze estimation technique is valid for purely visible light setups. The reported accuracy of around 0.40° shows the promise of the proposed technique.

Ono et al. [45] calculate the decomposition of the eye image, which takes into account variations caused by gaze direction, base eye appearance, and shifts in image cropping. Using this decomposition, they can encounter the most similar 3 training samples and use LLI to calculate the gaze with 2.4° accuracy.

Sugano et al. [46] use an LLI technique that allows head movements. They cluster the eye images according to the corresponding head pose and choose samples for interpolation only from the cluster with the same head pose as the current sample. Their system keeps learning from user interaction (i.e., mouse clicks) and continuously updates its parameters, adding clusters for new head poses when necessary. The reported average error is in the range 4-5°. The extended version of the work [47] provides methods for refining gaze labels acquired through mouse clicks, discarding high-error training samples, and locating the eye position better, thus decreasing the average error to only 2.9°.

Lu et al. [7, 29] decompose the gaze estimation problem into subproblems: (1) estimation under fixed head pose and (2) compensation of errors caused by head rotation and eye appearance distortion. Unlike other work, they do not choose the most similar local training samples explicitly; however, they argue that their method for weighting all the training samples automatically selects a small number of local samples. By learning eye appearance distortion from 5-second video clips and applying both compensations, they decrease the average error from 6° to 2.38° (and from 13.72° to 2.11° in the 2014 paper). In their later work [48, 49], instead of video clips (containing around 100 frames), they acquire only 4 additional training samples under reference head poses and synthesize extra training samples by modeling the change in eye appearance.

Alnajar et al. [50] propose a calibration-free estimation based on the assumption that humans have similar gaze patterns for the same stimulus. Here, first initial gaze points are calculated for a user without calibration, and then a transformation is calculated to map the user's gaze pattern to other users. For the initial gaze estimation, they either use the closest neighbors from the training set to reconstruct the current eye appearance (with samples from other users) or project the eye appearance to a 2D manifold to get the most similar samples.

Lai et al. [8] use random forests to learn the neighborhood structure for their joint head pose and eye appearance feature (HPEA). Gaze is estimated with linear interpolation using the neighbors in the random forest, yielding an accuracy of around 4.8° (horizontal and vertical combined).

Sugano et al. [51] build a multiview dataset and use it to reconstruct part of the face in 3D. They use this 3D model to generate synthetic samples acquired from different camera angles and use the extended dataset to train a random forest. Here, unlike their previous work [46], they do not divide the data strictly according to the head pose; however, they build sets of regression trees with overlapping head pose ranges (i.e., samples from a single head pose are used in building several sets of trees). Gaze is calculated as the average result from the nearest regression forests according to head pose, resulting in an average error of 6.5° with cross-subject training.

#### 3.1.3. Gaussian Processes

Gaussian process (GP) is another choice for the mapping in some gaze tracking methods. GP predictions are probabilistic and allow calculation of confidence intervals for the outputs which may be used as an indicator to detect when the calibration is no longer valid for the test data [20, 52].

Nguyen et al. [37, 53] describe a system where they use a Viola and Jones [15] eye detector and optical flow (OF) to detect and track the eye in the camera image. Then, the extracted eye image is fed to a GP to calculate the gaze point. In the extended work [37], they show that when the calibration is repeated in several head poses, the system can even become head pose invariant.

Ferhat et al. [9] also propose a similar method, where they use several Viola-Jones detectors (face, eye, nose, and mouth) to choose 8 anchor points on the face automatically and use the extracted eye image to train a GP. In the final system, the average error is 2° (horizontal and vertical combined).

Sugano et al. [10] use saliency information to automatically calibrate a gaze tracker while the subject is watching a video clip. While calibrating the GP-based tracker, instead of using known gaze positions, they train the GP with gaze probability maps calculated by aggregating several saliency maps.

### 3.2. Feature-Based Methods

In the appearance-based methods, the inputs to the mapping functions were the same across all techniques; therefore, we categorized them according to the mapping functions they used. However, in feature-based methods, the main difference is their feature set, and our categorization also reflects this difference.

#### 3.2.1. Anchor Point Position-Based Features

In this first subcategory of feature-based methods, the positions of important anchor points inside and around the eye (e.g., pupil (iris) center, inner and outer eye corners, and nostrils) are used as features. In some cases, they constitute distinct dimensions of the feature set, whereas in other cases, the relation between them (i.e., the vector connecting two anchor points) is used as the feature.

*Pupil Center-Eye Corner Vector*. In infrared gaze trackers, a feature widely used for gaze estimation is the pupil center-corneal reflection vector (PC-CR) [39]. The equivalent of this in natural light methods is the pupil center-eye corner vector (PC-EC) (or, alternatively, iris center-eye corner (IC-EC) vector).

The first use of the PC-EC vector in natural light eye trackers is proposed by two distinct research groups around the same time [20, 22, 54]. Hansen et al. [20, 54] use Active Appearance Model (AAM) and mean shift to track the eyes over time and find the positions of pupil center and eye corners. Gaze estimation is done by training a Gaussian process (GP) where the input is the PC-EC vector. The system results in an average error of around 1.6°, and the eye tracker is verified in an eye-typing interface. Zhu and Yang [22], on the other hand, propose methods for detecting the iris center and the eye corner with subpixel accuracy. They use a 2D linear mapping to estimate gaze positions from the feature vectors. They report an accuracy of around 1.2° from their experiments.

Valenti et al. [24, 55] propose a novel eye corner locator and combine it with a state-of-the-art eye center locator to calculate the EC-PC vector. Inspired by Zhu and Yang [22], they also use a 2D linear mapping for gaze estimation. In their later work [56], they make use of a head pose estimator and use the calculated transformation matrix to normalize the eye regions. The more accurate eye location found this way, in turn, is used to better estimate the head pose in a feedback loop. To solve the gaze estimation problem with head movements, they* retarget* the known calibration points to the monitor coordinates whenever there is a change in the head pose and calibrate the system again. With these improvements, they achieve average errors of between 2° and 5° in two experimental tasks.

Sesma et al. [39] normalize the PC-EC vector, dividing the vector components by the Euclidean distance between the inner and outer eye corners. For gaze estimation, they use both PC-EC vectors for the inner and outer eye corners and their experiments show the average error to be 1.25° when the head movement is constrained and around 3° when no chin rest is used.

Baek et al. [57] apply image rectification to rectify the eye images to a front facing head pose and combine it with a novel iris center localization method. They use second-order polynomial equations (as in [39]) to calculate the gaze and measure an accuracy of 2.42°.

Cheung and Peng [18] fit Active Shape Models (ASM) on images normalized using local sensitive histograms. With the novel methods they propose for iris center and eye corner detection, they achieve errors of 1.28° with fixed head pose and 2.27° with head movements.

*Others*. Some feature-based methods making use of anchor point positions may take a different path and combine or replace the EC and PC positions with information coming from other anchor points (e.g., nostrils) or simply calculate the features in another way.

In his thesis, Bäck [21] uses several geometrical features such as iris center, eye corner, nostril positions, head angle, and eye angles to create a rich feature vector and trains a NN for gaze estimation. The system is not tested heavily; however, the accuracy is reported to be in the range 2–4° and sometimes even up to 7-8°.

Torricelli et al. [23, 58] calculate several distance and angle features from both eyes to fill the feature vector. These features include distances of inner and outer eye corners to the iris center, the slopes of the lines connecting these points, and the positions of outer eye corners. The trained NN gaze estimation component results in average errors in the range 1-2°.

Ince and Kim [59] track the iris with a custom method and calculate the gaze using the iris center displacement between subsequent camera frames. The proposed system has an accuracy of 3.23° (horizontal and vertical combined). Nguyen et al. [60] take a similar approach and make use of the center-bias effect, which states that gaze distribution is biased towards the center of the screen [61]. Their system does not require any calibration and works by calculating the mean iris center over time and estimating the gaze through the difference of current iris center and the mean. The combined error in *x* and *y* directions is 3.43° of visual angle.

Wojciechowski and Fornalczyk [62] preprocess the eye images by calculating the edges and then extract their features which are the geometric center and center of mass of edge pixel positions. The final feature is the vector connecting these two locations (GC-CM), which is used to calculate the gaze estimation using the weighted average of data from 4 training points. The system has around 1.5° accuracy (combined).

Skodras et al. [17] track several moving and stationary anchor points (e.g., eye corner, eyelid control points, and iris center) and calculate vectors from their relative positions to build the final feature vector. They use linear regression for mapping to gaze point and achieve an accuracy of 2.96° (combined).

#### 3.2.2. Intensity-Based Features

In some feature-based methods, the direct connection between the image pixel intensity and feature vector is not broken completely. Williams et al. [14] combine the image pixel intensities with edge energies in their feature vector. They train a sparse, semisupervised Gaussian process (S^3^GP) which also infers the missing labels in the partially labeled training data. They make use of the confidence value for the GP to filter the estimation over time using a Kalman filter and achieve a final accuracy of 0.83°.

Lu et al. [28, 63] propose extracting 8D or 15D intensity features from the eye region, which is identical to resizing the grayscale eye image to 2 × 4 or 3 × 5 pixels, respectively. Together with the proposed subpixel alignment method for eye region, and adaptive linear regression (ALR) for gaze estimation, they can estimate the gaze point with up to 0.62° accuracy.

Xu et al. [31] extend the work of Lu et al. [28, 63] to increase the feature dimension to 120D (2 eye images of 6 × 10 pixels) and to use ridge regression for gaze estimation and achieve slightly worse results (1.06°).

#### 3.2.3. Traditional Computer Vision Features

Computer vision (CV) tasks such as object detection and classification are normally solved by using features (e.g., histogram of oriented gradients (HOG) [25], scale-invariant feature transform (SIFT) [64], and local binary patterns (LBPs) [27]) extracted around salient points in the images. However, until recently, this approach was still unexplored for the gaze tracking problem.

Martinez et al. [26] introduce this concept in a head mounted tracker, where they extract multilevel HOG features from eye images and use support vector regression (SVR) or relevance vector regression (RVR) to map these features to the gaze point, and achieve an accuracy of 2.20° (combined).

Zhang et al. [36] combine several features to build their feature vectors: color, pixel intensity, orientation (from several Gabor filters), Haar-like features, and spatiogram features (combining color histogram with spatial information). After generating this rich representation, they apply a dimensionality reduction technique to reduce the feature vector size to 50 and train a NN for gaze estimation. Although the reported average error is not very low (around 3.70°, when combined), the work is a great example of applying the traditional CV pipeline to gaze trackers.

Liang et al. [11] build on the previously explained S^3^GP technique [14] and train it with CS-LBP features [65], which is based on LBPs. They make use of spectral clustering to learn from partially labeled data and report an average error of 0.92°.

Schneider et al. [66] explore several feature types (DCT, LBP, and HOG) in conjunction with many alternatives for regression (GP, *k*-nearest neighbors (*k*NN), regression trees, SVR, RVR, and splines). They use a* dually supervised embedding* method to reduce the feature dimensionality, resulting in up to 31.2% decrease in the errors (best accuracy being 2.69° with 16-dimensional features based on HOG and LBP). Huang et al. [67] also take the same approach and review several feature types (LOG, LBP, HOG, and mHOG) and regression components (*k*NN, RF, GP, and SVR). They report that random forests (RF) combined with multilevel HOG (mHOG) features prove to be the most efficient combination (3.17° error) in a very challenging scenario (i.e., tablet computers), with free head movements.

Lately, convolutional neural networks (CNNs) are very popular in computer vision research, and Zhang et al. [13] are the first to use them for gaze tracking. CNN methods generally require a large dataset, and in their work they also present their dataset [68] which contains more than 200,000 images. They calculate features using a CNN and combine these features with the head pose information to build the complete feature vector. After testing with several regression functions (random forests, *k*NN, ALR, and SVR), the best accuracy they achieve is around 6°.

#### 3.2.4. Others

Ferhat et al. [30] use the segmented iris area to calculate their proposed features. In their feature vector (which contains 192 dimensions for an eye image of size 128 × 64), a given feature dimension holds the number of segmented pixels in the corresponding row or column of the iris segmentation image. Their system makes use of GP for regression and has an accuracy of 2.23° (combined).

### 3.3. Model-Based Methods

The models used in model-based gaze estimation methods are roughly divided into two: iris contour models (also known as one-circle algorithm), where an ellipse is fitted around the iris region, and eyeball models, where the main objective is to estimate the location of the eyeball center.

#### 3.3.1. Iris Contour Models

The direct least squares method for fitting ellipses onto a set of points [69] is influential in the development of iris contour models for gaze estimation. This method, complemented with the observation that the circular iris boundary appears as an ellipse in camera images, has enabled the development of several gaze tracking techniques.

Wang et al. [32, 70] develop the one-circle algorithm where they use edge detection to find pixels belonging to the iris boundary, and they fit an ellipse to this set of locations. Then, the ellipse is back-projected to the 3D space to find the iris contour circle, and its normal vector is used as the gaze vector. Their system has an average error of around 1°.

Hansen and Pece [71, 72] use an active contour method to track the iris edges over time, and (probably) using the one-circle method, their system estimates the gaze with around 4° accuracy.

Wu et al. [73] propose an extension with their two-circle algorithm, where they assume the elliptic iris contours for both eyes lie on the same plane or on parallel planes in 3D. With this assumption, they are able to estimate the gaze direction without the need for camera calibration.

Huang et al. [74] use randomized Hough transformation for iris contour fitting, whereas Zhang et al. [75] propose an improved RANSAC algorithm. The reported that accuracy for the latter work is 0.8° in a single direction.

Fukuda et al. [76] propose subpixel methods for iris contour estimation in low resolution images, achieving a combined average error of 3.35°. Mohammadi and Raie [77] train a support vector machine (SVM) to filter out the unrelated edge segments before applying the ellipse fitting, yielding an accuracy of 3.48°.

Wood and Bulling [12] detect the edges belonging to the iris from the image's radial derivative. After fitting the ellipse using the RANSAC method, the gaze estimation has an accuracy of 6.88°.

#### 3.3.2. Eyeball Models

Eyeball model-based techniques try to infer the eyeball center position and calculate the gaze vector as the line connecting this point with the iris center.

Ishikawa et al. [34] use an AAM to track the face and use the eye corner positions and the scale of the face to infer the anatomical constants for the user (i.e., eye geometry). This calibration is followed by iris detection by template matching and edge-based iris refinement to calculate the center of the iris. The geometrically calculated gaze has an average error of 3.2°.

Wu et al. [40] track the iris contours and the eyelids with a particle filter (PF) and use several appearance metrics to calculate the likelihood of a given particle (candidate). Experimental results show the mean error to be greater than 3.5°.

Xie and Lin [78] infer the position of the eyeball center and other personal parameters using a simple one-target calibration. They calculate the gaze geometrically by using the iris center and eye corner positions on the image, with 2° accuracy in a single direction.

Chen and Ji [33] use a generic face model that includes several facial anchor points (nostrils, inner and outer eye corners) and one of the eyeball centers. After calibrating for the personal parameters, they track the facial points and fit the 3D model to estimate the gaze with 2.7° accuracy.

Yamazoe et al. [19, 79] segment the eye image pixels into three classes: skin, sclera, and iris. Using the segmentation results, they calculate the most possible eye pose by minimizing the projection errors for a given candidate. The accuracy of the system is reported to be around 9°.

Reale et al. [41] use the detected iris contours to calculate the eyeball center, and after calibrating for the visual axis-optical axis shift and the eyeball radius, they estimate the gaze direction. Finally, the most recent work in this category is from Heyman et al. [80], who employ canonical correlation analysis (CCA) to estimate the head pose in a similar manner to AAMs. They calibrate the eyeball radius during initialization and estimate the iris center using a segmentation method. Their system estimates the gaze direction with 5.64° accuracy.

## 4. Calibration Strategies

Traditionally, calibration of the eye trackers consists of asking the subject to look at several targets in known positions. In this way, either the personal parameters (e.g., angle between visual and optical axis of the eye, eyeball radius) or the camera parameters (e.g., focal length, position with respect to the display) are learned.

Several papers that we analyze in this work present novel techniques to make this process easier for the subject using the tracker. Yamazoe et al. [19, 79] employ a transparent calibration process, where the user does not need to be aware at all. They track the face over time to construct the 3D model of the face and eyes and start calculating the gaze when the calibration is ready. Alnajar et al. [50] use other users' gaze patterns to help estimate the current user's patterns. Sugano et al. [10] completely remove the need for training data and estimate the gaze in a probabilistic manner using computed saliency maps.

Another approach to collecting the training data without needing special actions from the user is to let the user operate the computer normally and take samples during mouse clicks [13, 46, 47]. This method is based on the assumption that the user looks around the mouse pointer while clicking.

Head movements constitute a challenge for eye tracker calibration, and even small movements may cause large errors in the estimations of a calibrated tracker. This holds true especially for appearance-based gaze trackers. Valenti et al. [56] solve this problem by* retargeting* the calibration targets' positions to user's new field of view and calibrating the system again. Lu et al. [7, 29] require the user to record 5-second video clips while moving her/his head and use these to correct errors caused by head movements. Xie and Lin [78] require just a single target calibration, where the user keeps looking at the same position on the screen and moves her/his head around. Zhang et al. [13] take an approach based on large datasets and use other people's training data to calibrate a more accurate tracker.

Making the calibration process transparent for the user and collecting the required large amount of data are two conflicting objectives. In order to use the available training data to full extent, Williams et al. and Liang et al. [11, 14] use partially labeled data and annotate some of the unlabeled samples automatically. Ono et al. [45] create new samples by adding shifts while cropping the eye images, and in this way they can model the resulting appearance change and compensate for it while searching local samples. Lu et al. [48, 49] create synthetic training data by modeling the pixel flow around the eyes, whereas Sugano et al. [51] use 8 cameras to model a large part of the face in 3D and to generate training samples from previously unobserved head poses.

## 5. Dealing with Head Pose

Model-based visible light gaze tracking methods are normally invariant to head movements, assuming the preprocessing steps such as eye localization or model fitting do not fail. However, the same does not hold for the appearance-based and feature-based systems. As Lu et al. [29] demonstrate, the head movement not only adds a shift to the gaze angle, but also makes the calibration invalid by distorting the eye appearance for appearance-based methods.

The naive approach to solving the problem of head movements is adding more training data. Nguyen et al. [37, 53] propose repeating the calibration up to 10 times, while Lai et al. [8] require 34,000 training samples per user.

Zhang et al. [13] use a large dataset of previously collected images to train a feature-based gaze tracker. Here, training data collected from many subjects can be used in estimating the gaze for another person. Head pose invariance is achieved by incorporating the head pose angles into the feature set.

In other approaches [46, 47], the multipose training data is grouped according to head pose, and only a subset corresponding to the most similar head pose is used in the active calibration. To reduce the need for additional training data, Lu et al. [48] synthetically generate training samples for unseen head poses.

Instead of pouring more data into the system, another option is to apply compensations or small fixes to keep the current calibration working. Lu et al. [63] propose an eye image alignment scheme to undo the deformation in these images. In their other works [7, 29], they train regression for this task and combine it with a compensation for head rotation.

Valenti et al. [56] keep the calibration targets in a flexible representation and* retarget* these to the display coordinates whenever the head pose is changed and recalibrate their system.

Cheung and Peng [18] assume the PC-EC feature is completely invariant to head pose and apply only head rotation compensation in their system.

## 6. Available Datasets

Several papers that we analyzed contain a summary of publicly available datasets for visible light gaze tracking [13, 51, 81]. However, they are mostly for the purpose of comparison with the presented datasets in the mentioned work and thus may lack some pieces of related information.

In Table 2, we bring together all the datasets mentioned in these works (with several more recently published additions), in an attempt to provide a reference for future research in the field.

One of the datasets [82] cited in the previous reviews has been removed, as it provided data for a head mounted setup.

## 7. Gaze Tracking as a Service

**Figure 2:**
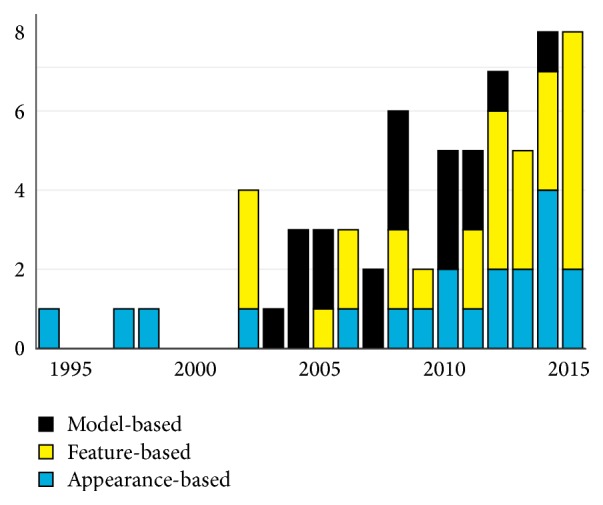
Number of works from different categories of eye trackers according to the publication year.

While visible light gaze tracking has become a hot topic in the academia in recent years (as can be observed in Figure 2), the industry is not trailing far behind either. Here, we talk about several companies already providing gaze tracking service based on regular cameras found on consumer devices.

GazeHawk [83] (now closed) was enabling its customers to convey remote eye tracking studies inside the user's browser. xLabs [84] is another similar service, which is also available as a Chrome extension. With the extension, several demos (including continuous calibration by an ant smashing game) can be tried. Lastly, Sticky [85] also provides a JavaScript-based service, suggesting use cases such as online ad placement and web page optimization. As the only service with detailed specifications, their eye tracker provides an average accuracy of 2.4°.

Other possible clients for this type of eye tracker are the game or application developers. SentiGaze [86] provides an SDK for developers targeting the Windows platform. FaceTrack from Visage Technologies [87] provides a similar C++ SDK for developers, with augmented reality, view control in gaming, and view-dependent rendering suggested as possible use cases. The SDK provides detailed information such as mouth contour, chin pose, and eye openness, in addition to the gaze information. InSight SDK [88] takes one step further and combines the gaze information with mood, age, and gender estimation.

With the transition from desktop programs to mobile apps in recent years, two companies see a possibility for gaze tracking on this platform. Snapdragon [89] provides an SDK for Android apps, whereas Umoove [90] has a product on both iOS and Android platforms.

## 8. Open Source Projects

A few works that we analyze in this paper have released their source code with an open source license. In this section, we list these options so that new projects in the field will have a starting point for the codebase.  Table 3 shows a summary of the listed projects.

Opengazer [91] is an eye tracker from Cambridge University, which is unfortunately no longer maintained. It uses Gaussian process regression with eye images as features, which is similar to the technique described by Nguyen et al. [37]. NetGazer [92] is the port of Opengazer for the Windows platform and is not maintained anymore either.

In the recent years, a fork of Opengazer project, named CVC Eye Tracker [93], was made available and is maintained actively by researchers from Universitat Autònoma de Barcelona. This project is the basis for two works analyzed in our review [9, 30].

Neural Network Eye Tracker (NNET) [94] is the NN-based eye tracker implementation for iPad devices, which is presented in two articles [43, 44]. EyeTab [95] is another open source codebase for tablet computers, which uses the iris contour model-based method described by Wood and Bulling [12].

Recently, the TurkerGaze project [31, 96] was made available on GitHub. This application is totally implemented in JavaScript (JS), which makes it platform independent (with possible extension to the mobile). The library has a polished interface for calibration and verification and comes with a small application for analyzing the gaze patterns recorded during conducted experiments. Although its proposed usage area is to enable crowdsourcing eye tracking tasks on platforms similar to Amazon Mechanical Turk, we believe it will have a larger impact on both academic works and web-based applications.

One last open source application is Camgaze [97], which is written in Python and calculates binocular gaze estimations.

## 9. Summary and Conclusions

In this work, we have tried to present a review of the state of the art in remote, natural light gaze trackers. Although in recent years many great works were published in the field, and the accuracy gap to reach the infrared-based trackers is closing, many open problems and unexplored approaches still remain.

Apart from the accuracy, the biggest challenges to these trackers are (a) making the calibration less painful for the user and (b) allowing free head movements. As we analyzed in the previous sections dedicated to these two problems, the field witnessed amazing works recently. Some open lines of work that we have identified in these areas are the following:

- *Maintaining Personal Calibration*. Most of the works we analyzed require some sort of calibration, be it for personal parameters for the user, for camera properties, or simply for training the gaze mapping component. Although some techniques may already allow it (without stating explicitly), reusing the calibration information for the subsequent sessions of the same user is still pending extensive analysis. With such a technique, calibration before each session can be simplified or removed altogether.
- *Using Calibration Data from Other Users*. Despite being explored in a few papers [13, 50], we believe the accumulation (or collection) of training data from people other than the current user will receive more focus in the coming years. This is analogous to training classifiers or detectors in other computer vision tasks, and it will let us make better use of the large datasets that we have begun to build.
- *Other Ways of Collecting Data*. Collecting calibration samples each time the user clicks the mouse enabled us to create very large datasets for the first time [13, 46, 47]. Especially with the advent of JavaScript-based eye trackers [31], other possibilities such as remotely crowdsourcing data collection will emerge. Larger data will eventually let us explore previously impossible ideas, a trend which is common in computer vision.

These lines of work are mostly around the topic of data collection and calibration, and they will help solve the large data needs of training for different head poses.

Most of the recent high-performing techniques [11, 14, 28, 63] are using feature-based gaze estimation, which shows the promise of this category over appearance- or model-based methods. Figure 2 also shows this tendency, and the increase in feature-based methods can be observed clearly. Over the next years, we will probably see more examples of similar work with the following focus points:

- *Different Features*. The PC-EC vector, pixel intensity and color, and other standard features (such as HOG and LBP) have been used so far. New feature representations that may be better suited to the problem at hand will greatly improve the eye tracking accuracy. The desired characteristics of such features are (a) invariance to head pose, (b) invariance to intensity changes, and (c) invariance to personal appearance differences.
- *Migrating Proven Ideas from Other CV Fields*. Use of convolutional neural networks (CNNs) [13], features such as HOG and LBP, and in general the computer vision (CV) pipeline [36] are changing our approach to the gaze tracking problem. These ideas were already commonplace in other areas of CV, and we believe our community will keep transferring insights which have been proven to work for other problems.

Apart from these technical challenges and lines of work, as a society, our biggest problems are related to transparency and letting others build on our work.

Firstly, only very few of these works report their accuracy on publicly available datasets or publish the dataset they use. This is a must in other computer vision areas so that the results from techniques can be compared and verified. Moreover, standardization of the processing pipeline will immediately follow (as it depends on the training data structure) and will foster our progress.

Our second problem is that only few works make their source code available. This prevents other researchers from* standing on the shoulders of giants* and hinders the rate of our progress. We believe that, by releasing our source code, we can create stronger ties and cooperation in the field.

In conclusion, the amount and quality of the recent work in the field are promising and signal even faster progress in the coming years. With this* map* of the current state of the art that you are holding in your hands (or gazing at through an electronic display), we hope to provide a reference point for all these amazing works we cannot wait to see.

## Figures and Tables

**Table 1:** Summary and results of all the techniques analyzed in this work. Methods are grouped into categories for easier reference. HP column shows whether the technique has head pose invariance or not. Techniques allowing small head movements are denoted by ≈ symbol. Output column shows what type of gaze is calculated: point of gaze (∘) or line of gaze (∡).

	Feature	Mapping	Calibration	HP	Dataset	Output	Accuracy	References	Comments
Appearance-based	—	NN	Grid	—	—	∘	1.5–4	[16, 35, 42–44]	
	—	GP	Grid	—	[98]	∘	2	[9]	
	—	GP	Grid	≈	—	∘	n/a	[37, 53]	Rigorous calib. for HP
	—	LLI	Grid	—	—	∘	0.4	[38]	IR to locate eye
	—	LLI	Grid	—	—	∘	2.4	[45]	
	—	LLI	Grid + HP	✓	—	∘	2.2–2.5	[7, 29, 48, 49]	0.85° error with fixed HP
	—	LLI	Grid	✓	—	∡	4.8	[8]	
	—	LLI	—	✓	—	∘	3–5	[46, 47]	Incremental calibration
	—	LLI	Grid	✓	[99]	∡	4	[51]	8 cameras
	—	LLI	—	—	—	∘	3.5–4.3	[10, 50]	Saliency for calibration

Feature-based	PC-EC	GP	Grid	—	—	∘	1.6	[20, 54]	
	PC-EC	LI	Grid	—	—	∡	1.2	[22]	
	PC-EC	LI	Grid	—	—	∘	n/a	[24, 55]	
	PC-EC	PI	Grid	—	—	∘	1.2	[39]	3° without chin rest
	PC-EC	LI	Grid	✓	—	∘	2–5	[56]	
	PC-EC	PI	Grid	—	—	∘	2.4	[57]	
	PC-EC	PI	Grid	✓	—	∘	2.3	[18]	1.2° error with fixed HP
	Several	NN	Grid	—	—	∘	1-2	[23, 58]	
	Several	NN	Grid	✓	—	∘	2–7	[21]	Few tests
	EC shift	n/a	Grid	—	—	∘	3.2	[59]	
	EC shift	LI	—	—	—	∘	3.4	[60]	Hand-coded params.
	GC-CM	LI	Grid	—	—	∘	1.5	[62]	
	Several	LI	Grid	—	—	∘	3	[17]	
	Edge energy	S^3^GP	Grid	—	—	∘	0.8	[14]	
	Intensity	ALR	Grid	≈	—	∘	0.6	[28, 63]	8D or 15D feats.
	Intensity	RR	Grid	—	—	∘	1.1	[31]	120D feats.
	HOG	SVR/RVR	Grid	—	—	∘	2.2	[26]	
	Several	NN	Grid	—	—	∘	3.7	[36]	Dim. reduced to 50
	CS-LBP	S^3^GP	Grid	—	—	∘	0.9	[11]	Partially labelled data
	Several	Several	Grid	—	[100]	∘	2.7	[66]	Dim. reduced to 16
	Several	Several	Grid	✓	[101]	∘	3.2	[67]	
	CNN	Several	Continuous	✓	[68]	∡	~6	[13]	Calib. from dataset
	Segmentation	GP	Grid	—	—	∘	2.2	[30]	

	Model		Calibration	HP	Dataset	Output	Accuracy	References	Comments

Model-based	Iris contour		Camera	✓	—	∡	1	[32, 70]	One-circle alg.
	Iris contour		Grid	✓	—	∘	4	[71, 72]	
	Iris contour		—	✓	—	∡	n/a	[73]	Two-circle alg.
	Iris contour		Camera	—	—	∘	n/a	[74]	
	Iris contour		Camera	—	—	∡	0.8	[75]	Error for single dir.
	Iris contour		Grid	✓	—	∡	3.3	[76]	
	Iris contour		Grid	✓	—	∡	3.5	[77]	
	Iris contour		Grid	✓	—	∘	6.9	[12]	
	Eyeball		Grid	✓	—	∡	3.2	[34]	Calib. personal params.
	Eyeball		Grid	—	—	∡	3.5	[40]	PF tracking
	Eyeball		1 target	✓	—	∡	~2	[78]	Error for single dir.
	Eyeball		Grid	✓	—	∘	2.7	[33]	
	Eyeball		—	✓	—	∡	9	[19, 79]	Autocalibration
	Eyeball		Grid	✓	—	∘	n/a	[41]	
	Eyeball		—	✓	[102, 103]	∡	5.6	[80]	

**Table 2:** Publicly available datasets for remote, natural light gaze tracking.

	Year	# subjects	# targets	# head poses	Calibration	Resolution	Dataset size	References
UUlm	2007	20	2–9	19	Yes	1600 × 1200	2,200 imgs.	[103, 104]
HPEG	2009	10	Continuous	2	Yes	640 × 480	20 videos (~6.6 k imgs.)	[102, 105]
Gi4E	2012	103	12	1	No	800 × 600	1,236 imgs.	[106–108]
CAVE	2013	56	21	5	Yes	5184 × 3456	5,880 imgs.	[81, 100]
CVC	2013	12	12–15	4	Yes	1280 × 720	48 videos (~20 k imgs.)	[9, 98]
EYEDIAP	2014	16	Continuous	Continuous	Yes	1920 × 1080	94 videos	[109, 110]
Multiview	2014	50	160	8 (+synthesized)	Yes	1280 × 1024	64,000 imgs. (+synth.)	[51, 99]
MPIIGaze	2015	15	Continuous	Continuous	No	1280 × 720	213,659 imgs.	[13, 68]
OMEG	2015	50	10	Continuous	No	1280 × 1024	44,827 imgs.	[111]
TabletGaze	2015	51	35	Continuous	No	1280 × 720	816 videos (~120 k imgs.)	[67, 101]

**Table 3:** Open source gaze trackers and the related publications.

	Language	Platform	License	References
Opengazer	C/C++	Linux/Mac	GPLv2	[91]
NetGazer	C++/C#	Windows	GPLv2	[92]
CVC ET	C/C++	Linux/Mac	GPLv2	[9, 30, 93]
NNET	Objective C	iOS	GPLv3	[43, 44, 94]
EyeTab	Python/C++	Windows	MIT	[12, 95]
TurkerGaze	JavaScript	All	MIT	[31, 96]
Camgaze	Python	All	?	[97]
